# Supplementary material for: Heat adaptation of phage T7 under an extended genetic code
Source: Virus Evol. 2021 Dec 1;7(2):veab100. doi: 10.1093/ve/veab100 (PMC8923235; doi:10.1093/ve/veab100)
Supplement: veab100_Supp [file veab100_supp.zip › Sequencing_Analysis.zip!/Sequencing_Analysis/S3/evidence/JC_SIDE_1_52.html]

BRESEQ :: Evidence


| New junction evidence | | | | | | | | | | | |
| --- | --- | --- | --- | --- | --- | --- | --- | --- | --- | --- | --- |
|  | | seq id | position | reads (cov) | reads (cov) | score | skew | freq | annotation | gene | product |
| \* | ? | NC\_001604.1 | = 16508 | 832 (0.480) | 277 (0.160) | 147/586 | 6.7 | 27.7% | intergenic (–/–) | *–/–* | –/– |
|  | ? | NC\_001604.1 | 16970 = | 637 (0.380) |  |  |  |  | intergenic (–/–) | *–/–* | –/– |
| Rejected: Coverage evenness skew score above cutoff. | | | | | | | | | | | |

Only 100 of 1324 total aligned reads displayed.  

`CTCTCATCTGCAAACTGTGGATTATCAAGACCGAAGAGATGCTCGTAGAGAAAGGCTTGAAGCATGGCTGGGATGGGGACTTTGCGTACATGGCATGGGTACATGATGAAATCCAAGTAGGCTGCCGTACCGAAGAGATTGCTCAGGTGGTCATTGAGACCGCACAAGAAGCGATGCGCTGGGTTGGAGACCACTGGAACTTCCGGTGTCTTCTGGATACCGAAGGTAAGATGGGTCCTAATTGGGCGATTTGCCACTGATACAGGAGGCTACTCATGAACGAAAGACACTTAACAGGTGCTGCTTCTGAAATGCTAGTAGCCTACAAATTTACCAAAGCTGGGTACACTGTCTATTACCCTATGCTGACTCAGAGTAAAGAGGACTTGGTTGTATGTAAGGATGGTAAATTTAGTAAGGTTCAGGTTAAAACAGCCACAACGGTTCAAACCAACACAGGAGATGCCAAGCAGGTTAGGCTAGGTGGATGCGGTAGGTCCGAATATAAGGATGGAGACTTTGACATTCTTGCGGTTGTGGTTGACGAAGATGTGCTTATTTTCACATGGGACGAAGTAAAAGGTAAGACATCCATGTGTGT  >  NC_001604.1/16208‑16808`  

                                                                                                                                                                                                                                                                                                            `|`                                                                                                                                                                                                                                                                                                              
    `cATCTGCAAACTGTGGATTATCAAGACCGAAGAGATGCTCGTAGAGAAAGGCTTGAAGCATGGCTGGGATGGGGACTTTGCGTACATGGCATGGGTACATGATGAAATCCAAGTAGGCTGCCGTACCGAAGAGATTGCTCAGGTGGTCATTGAGACCGCACAAGAAGCGATGCGCTGGGTTGGAGACCACTGGAACTTCCGGTGTCTTCTGGATACCGAAGGTAAGATGGGTCCTAATTGGGCGATTTGCCACTGATACAGGAGGCTACTCATGAACGAAAGACACTTAACAGGTgctgct                                                                                                                                                                                                                                                                                                          >  1:54773/1‑301 (MQ=255)`  
                  `ggATTATCAAGACCGAAGAGATGCTCGTAGAGAAAGGCTTGAAGCATGGCTGGGATGGGGACTTTGCGTACATGGCATGGGTACATGATGAAATCCAAGTAGGCTGCCGTACCGAAGAGATTGCTCAGGTGGTCATTGAGACCGCACAAGAAGCGATGCGCTGGGTTGGAGACCACTGGAACTTCCGGTGTCTTCTGGATACCGAAGGTAAGATGGGTCCTAATTGGGCGATTTGCCACTGATACAGTAGGCTACTCATGAACGAAAGACACTTAACAGGTGCTGCTTCTGAAATGCtagt                                                                                                                                                                                                                                                                                            >  1:180336/1‑301 (MQ=255)`  
                  `ggATTATCAAGACCGAAGAGATGCTCGTAGAGAAAGGCTTGAAGCATGGCTGGGATGGGGACTTTGCGTACATGGCATGGGTACATGATGAAATCCAAGTAGGCTGCCGTACCGAAGAGATTGCTCAGGTGGTCATTGAGACCGCACAAGAAGCGATGCGCTGGGTTGGAGACCACTGGAACTTCCGGTGTCTTCTGGATACCGAAGGTAAGATGGGTCCTAATTGGGCGATTTGCCACTGATACAGGAGGCTACTCATGAACGAAAGACACTTAACAGGtgcgattgtccccaatggtaa                                                                                                                                                                                                                                                                                            <  1:6165/301‑19 (MQ=255)`  
                      `tATCAAGACCGAAGAGATGCTCGTAGAGAAAGGCTTGAAGCATGGCTGGGATGGGGACTTTGCGTACATGGCATGGGTACATGATGAAATCCAAGTAGGCTGCCGTACCGAAGAGATTGCTCAGGTGGTCATTGAGACCGCACAAGAAGCGATGCGCTGGGTTGGAGCCCACTGGAACTTCCGGTGTCTTCTGGATACCGAAGGTAAGATGGGTCCTAATTGGGCGATTTGCCACTGATACAGGAGGCTACTCATGAACGAAAGACACTTAACAGGTGCTGCTTCTGAAATGCTAGTAGcc                                                                                                                                                                                                                                                                                        <  1:109097/301‑1 (MQ=255)`  
                           `aGACCGAAGAGATGCTCGTAGAGAAAGGCTTGAAGCATGGCTGGGATGGGGACTTTGCGTACATGGCATGGGTACATGATGAAATCCAAGTAGGCTGCCGTACCGAAGAGATTGCTCAGGTGGTCATTGAGACCGCACAAGAAGCGATGCGCTGGGTTGGAGACCACTGGAACTTCCGGTGTCTTCTGGATACCGAAGGTAAGATGGGTCCTAATTGGGCGATTTGCCACTGATACAGGAGGCTACTCATGAACGAAAGACACTTAACAGGtgcgattgtccccaatggtaaacagaagga                                                                                                                                                                                                                                                                                   <  1:122020/301‑28 (MQ=255)`  
                            `gACCGAAGAGATGCTCGTAGAGAAAGGCTTGAAGCATGGCTGGGATGGGGACTTTGCGTACATGGCATGGGTACATGATGAAATCCAAGTAGGCTGCCGTACCGAAGAGATTGCTCAGGTGGTCATTGAGACCGCACAAGAAGCGATGCGCTGGGTTGGAGACCACTGGAACTTCCGGTGTCTTCTGGATACCGAAGGTAAGATGGGTCCTAATTGGGCGATTTGCCACTGATACAGGAGGCTACTCATGAACGAAAGACACTTAACAGGTGCTGCTTCTGAAATGCTAGTAGCCTACaaa                                                                                                                                                                                                                                                                                  <  1:308562/301‑1 (MQ=255)`  
                                  `agagATGCTCGTAGAGAAAGGCTTGAAGCATGGCTGGGATGGGGACTTTGCGTACATGGCATGGGTACATGATGAAATCCAAGTAGGCTGCCGTACCGAAGAGATTGCTCAGGTGGTCATTGAGACCGCACAAGAAGCGATGCGCTGGGTTGGAGACCACTGGAACTTCCGGTGTCTTCTGGATACCGAAGGTAAGATGGGTCCTAATTGGGCGATTTGCCACTGATACAGGAGGCTACTCATGAACGAAAGACACTTAACAGGTGCTGCTTCTGAAATGCTAGTAGCCTACAAATTTAcc                                                                                                                                                                                                                                                                            <  1:280996/301‑1 (MQ=255)`  
                                         `cTCGTAGAGAAAGGCTTGAAGCATGGCTGGGATGGGGACTTTGCGTACATGGCATGGGTACATGATGAAATCCAAGTAGGATGCCGTACCGAAGAGATTGCTCAGGTGGTCATTGAGACCGCACAAGAAGCGATGCGCTGGGTTGGAGACCACTGGAACTTCCGGTGTCTTCTGGATACCGAAGGTGAGATGGGTCCTAATTGGGCGATTTGCCACTGATACAGGAGGCTACTCATGAACGAAGGACACTTAACAGGTGCTGCTTCTGAAATGCTAGTAGCTTACAAATTTACCCAAGCTg                                                                                                                                                                                                                                                                     >  1:65541/1‑301 (MQ=255)`  
                                            `gTAGAGAAAGGCTTGAAGCATGGCTGGGATGGGGACTTTGCGTACATGGCATGGGTACATGATGAAATCCAAGTAGGCTGCCGTACCGAAGAGATTGCTCAGGTGGTCATTGAGACCGCACAAGAAGCGATGCGCTGGGTTGGAGACCACTGGAACTTCCGGTGTCTTCTGGATACCGACGGTAAGATGGGTCCTAATTGGGCGATTTGCCACTGATACAGGAGGCTACTCATGAACGAAAGACACTTAACAGGTGCTGCTTCTGAAATGCTAGTAGCCTACAAATTTACCAAATCTGGGt                                                                                                                                                                                                                                                                  >  1:189495/1‑301 (MQ=255)`  
                                              `gatgggtataagagacaggggCTGGGATGGGGACTTTGCGTACATGGCATGGGTACATGATGAAATCCAAGTAGGCTGCCGTACCGAAGAGAGTGCTCAGGTGGTCATTGAGACCGCACAAGAAGCGATGCGCTGGGTTGGAGACCACTGGAACTTCCGGTGTCTTCTGGATACCGAAGGTAAGATGGGTCCTAATTGGGCGATTTGCCACTGATACAGGAGGCTACTCATGAACGAAAGACACTTAACAGGTGCTGCTTCTGAAATGCTAGTAGCCTACAAATTTACCAAAGCTGGGTac                                                                                                                                                                                                                                                                <  1:242900/282‑1 (MQ=255)`  
                                               `gagaAAGGCTTGAAGCATGGCTGGGATGGGGACTTTGCGTACATGGCATGGGTACATGATGAAATCCAAGTAGGCTGCCGTACCGAAGAGATTGCTCAGGTGGTCATTGAGACCGCACAAGAAGCGATGCGCTGGGTTGGAGACCACTGGAACTTCCGGTGTCTTCTGGATACCGAAGGTAAGATGGGTCCTAATTGGGCGATTTGCCACTGATACAGGAGGCTACTCATGAACGAAAGACACTTAACAGGTGCTGCTTCTGAAATGCTAGTAGCCTACAAATTTACCAAAGCTGGGTaca                                                                                                                                                                                                                                                               >  1:68225/1‑301 (MQ=255)`  
                                                     `ggtatgaatgaTGGCTGGGATGTGGAGTTTGCGTACATGGCATGTGTACAAGAAGAAATCCAAGTAGGCTGCCGTACCGAAGAAATTGCGAAGGTGGTCATTGAGACCGCACAAGAAGCGATGCGCTGAGTTGGAGACCACTGGAGCTTCCGGTGTCTTCTGGATACCGAAGTTAAGATGGGTCCTAATTGGGCGATTTGCCACTGATACAGGAGGCTACTCATGAACTAAAGACACTTAACAGGTGCTGCTTCTGAAATGCTAGTAGCCTACAAATTTACCAAAGCTGGGTACACTGTCt                                                                                                                                                                                                                                                         <  1:305698/291‑1 (MQ=255)`  
                                                     `ggCTTGAAGCATGGCTGGGATGGGGACTTTGCGTACATGGCATGGGTACATGATGAAATCCAAGTAGGCTGCCGTACCGAAGAGATTGCTCAGGTGGTCATTGAGACCGCACAAGAAGCGATGCGCTGGGTTGGAGACCACTGGAACTTCCGGTGTCTTCTGGATACCGAAGGTAAGATGGGTCCTAATTGGGCGATTTGCCACTGATACAGGAGGCTACTCATGAACGAAAGACACTTAACAGGTGCTGCTTCTGAAATGCTCGTATCATACAAATTTACCAAAGCTGGGTCCACTGTCt                                                                                                                                                                                                                                                         >  1:170948/1‑301 (MQ=255)`  
                                                      `gCTTGAAGCATGGCTGGGATGGGGACTTTGCGTACATGGCATGGGTACATGATGAAATCCAAGTAGGCTGCCGTACCGAAGAGATTACTCAGGTGGTCATTGAGACCGCACAAGAAGCGATGCGCTGGGTTGGAGACCACTGGAAATTCCGGTGTCTTCTGGATACCGAAGGTAAGATGGGTCCTAATTGGGCGATTTGCCACTGATACAGGAGGCTACTCATGAACGAAAGACACTTAACAGGTGCTGCTTCTGAAATGCTAGTAGCCTACAAATTTACCAAAGCTGGGTACACTGTCTa                                                                                                                                                                                                                                                        >  1:258441/1‑301 (MQ=255)`  
                                                          `gAAGCATGGCTGGGATGGGGACTTTGCGTACATGGCATGGGTACATGATGAAATCCAAGTAGGCTGCCGTACCGAAGAGATTGCTCAGGTGGTCATTGAGACCGCACAAGAAGCGATGCGCTGGGTTGGAGACCACTGGAACTTCCGGTGTCTTCTGGATACCGAAGGTAAGATGGGTCCTAATTGGGCGATTTGCCACTGATACAGGAGGCTACTCATGAACGAAAGACACTTAACAGGTGCTGCTTCTGAAATGCTAGTAGCCTACAAATTTACCAAAGCTGGGTACACTGTCTATTAc                                                                                                                                                                                                                                                    <  1:186785/301‑1 (MQ=255)`  
                                                          `gAAGCATGGCTGGGATGGGGACGTTGCGTACATGGCATGGGTACATGATGAAATCCAAGTAGGCTGCCGTACCGAAGAGATTGCTCAGGTGGTCATTGAGACCGCACAAGAAGCGATGCGCTGGGTTGGAGACCACTGGAACATCCGGTGTCTTCTGGATACCGAAGGTAAGATGGGTCCTAATTGGGCGATTTGCCACTGATACAGGAGGCTACTCATGAACGAAAGACACTTAACAGGTGCTGCTTCTGAAATGCTAGTAGCCTACAAATTTACCAAAGCTGGGTACACTGTCTATTAc                                                                                                                                                                                                                                                    <  1:156754/301‑1 (MQ=255)`  
                                                          `aaaGCATGGCTGGGATGGGGACTTTGCGTACATGGCATGGGTACATGATGAAATCCAAGTAGGCTGCCGTACCGAAGAGAATGCGCAGGTGGTCATTGAGACCGCACAAGAAGCGATGCGCTGGGTTGGAGACCACTGGAACTTCCGGTGTCTTCTGGATACCGAAGGTAAGATGGGTCCTAATTGGGCGATTTGCCACTGATACAGGAGGCTACTCATGAACGAAAGACACTTAACAGGTGCTGCTTCTGAAATGCTAGTAGCCTACAAATTTACCAAAGCTGGGTACACTGTCTATTAc                                                                                                                                                                                                                                                    <  1:248542/300‑1 (MQ=255)`  
                                                           `aaGCATGGCTGGGATGGGGACTTTGCGTACATGGCATGGGTACATGATGAAATCCAAGTAGGCTGCCGTACCGCAGAGATTGCTCAGGTGGTCATTGAGACCGCACAAGAAGCGATGCGCTGGGTTGGAGACCACTGGAACTTCCGGTGTCTTCTGGATACCGAAGGTAAGATGGGTCCTAATTGGGCGATTTGACACTGATACAGGAGGCTACTCATGAACGAATGACACTTAACAGGTGCTGCTTCTGAAATGCTAGTAGCCTACAAATTTACCAAATCTGGGTACACTGTCTATTAcc                                                                                                                                                                                                                                                   >  1:194177/1‑301 (MQ=255)`  
                                                            `aGCATGGCTGGGATGGGGACTTTGCGTACATGGTATGGGTACATGATGAAATCCAAGTAGGCTGCCGTACCGAAGAGATTGCTCAGGTGGTCATTGAGACCGCACAAGAAGCGATGCGCTGGGTTGGAGACCACTGGAACTTCCGGTGTCTTCTGGATACCGAAGGTAAGATGGGTCCTAATTGGGCGATTTGCCACTGATACAGGAGGCTACTCATGAACGAAAGACACTTAACAGGTGCTGCTTCTGAAATGCTAGTAGCCTACAAATTTACCAAAGCTGGGTACACTGTCTATTAccc                                                                                                                                                                                                                                                  <  1:95816/301‑1 (MQ=255)`  
                                                             `gCATGGCTGGGATGGGGACTTTGCGTACATGGCATGGGTACATGATGAAATCCAAGTAGGCTGCCGTACCGAAGAGATTGCTCAAGTGGTCATTGAGACCGCACAAGAAGCGATGCGCTGGGTTGGAGACCACTGGAACTTCCGGTGTCTTCTGGATACCGAAGGTAAGATGGGTCCTAATTGGGCGATTTGCCACTGATACAGGAGGCTACTCATGAACGAAAGACACTTAACAGGTGCTGCTTCTGAAATGCTAGTAGCCTACAAATTTACCAAAGCTGGGTACACTGTCTATTACCCt                                                                                                                                                                                                                                                 >  1:183718/1‑301 (MQ=255)`  
                                                                 `ggatGGGATGGGGACTTTGCGTACATGGCATGGGTACATGATGAAATCCAAGTAGGCTGCCGTACCGAAGAGATTGCTCAGATGGTCATTGAGACCGCACAAGAAGCGATGCGCTGGGTTGGAGACCACTGGAACTTCCGGTGTCTTCTGGATACCGAAGGTAAGATGGGTCCTAATTGGGCGATTTGACACTGATACAGGAGGCTACTCATGAACGAAAGACACTTAACAGGTGCTGCTTCTGAAATGCTAGTAGCCTACAAATTTACCAAAGCTGGGTACACTGTCTATTACCCTATGc                                                                                                                                                                                                                                             <  1:79021/298‑1 (MQ=255)`  
                                                                      `ggATGGGGACTTTGCGTACATGGCATGGGTACATGATGAAATCCAAGTAGGCTGCCGTACCGAAGAGATTGCTCAGGTGGTCATTGAGACCGCACAAGAAGCGATGCGCTGGGTTGGAGACCACTGGAACTTCCGGTGTCTTCTGGATACCGAAGGTAAGATGGGTCCTAATTGGGCGATTTGCCACTGATACAGGAGGCTACTCATGAACGAAAGACACTTAACAGGTGCTGCTTCTGAAATGCTAGTAGCCTACAAATTTACCAAAGCTGGGTACACTGTCTATTACCCTATGCTGACt                                                                                                                                                                                                                                        <  1:262574/301‑1 (MQ=255)`  
                                                                       `gATGGGGACTTTGCGTACATGGCATTGGTACATGATGAAATCCAAGTAGGCTGCCGTACCGAAGAGATTGCTCAGGTGGTCATTGAGACCGCACAAGAAGCGATGCGCTGGGTTGGAGACCACTGGAACTTCCGGTGTCTTCTGGATACCGAAGGTAAGATGGGTCCTAATTGGGCGATTTGCCACTGATACAGGAGGCTACTCATGAACGAAAGACACTTAACAGGTGCTGCTTCTGAAATGCTAGTAGCCTACAAATTTACCAAAGCTGGGTACACTGTCTATTACCCTATGCTGACTc                                                                                                                                                                                                                                       <  1:294461/301‑1 (MQ=255)`  
                                                                       `gATGGGGACTTTGCGTACATGGCATGGGTACATGATGAAATCCAAGTAGGCTGCCGTACCGAAGAGATTGCTCAGGTGGTCATTGAGACCGCACAAGAAGCGATGCGCTGGGTTGGAGACCACTGGAACTTCCGGTGTCTTCTGGATACCGAAGGTAAGATGGGTCCTAATTGGGCGATTTGCCACTGATACAGGAGGCTACTCATGAACGAAAGACACTTAACAGGTGCTGCTTCTGAAATGCTAGTAGCCTACAAATTTACCAAAGCTGGGTACACTGTCTATTACCCTATGCTGACTc                                                                                                                                                                                                                                       <  1:204041/301‑1 (MQ=255)`  
                                                                       `gATGGGGACTTTGCGTACATGGCATGGGTACATGATGAAATCCAAGTAGGCTGCCGTACCGAAGAGATTGCTCAGGTGGTCATTGAGACCGCACAAGAAGCGATGCGCTGGGTTGGAGACCACTGGAACTTCCGGTGTCTTCTGGATACCGAAGGTAAGATGGGTCCTAATTGGGCGATTTGCCACTGATACAGGAGGCTACTCATGAACGAAAGACACTTAACAGGTGCTGCTTCTGAAATGCTAGTAGCCTACAAATTTACCAAAGCTGGGTACACTGTCTATTACCCTATGCTGACTc                                                                                                                                                                                                                                       >  1:317588/1‑301 (MQ=255)`  
                                                                       `gATGGGGACTTTGCGTACATGGCATGGGTACATGATGAAATCCAAGTAGGCTGCCGTACCGAAGAGATTGCTCAGGTGGTCATTGAGACCGCAAAAGAAGCGATGCGCTGGGTTGGAGACCACTGGAACTTCCGGTGTCTTCTGGATACCGAAGGTAAGATGGGTCCTAATTGGGCGATTTGCCACTGATACAGGAGGCTACTCATGAACGAAAGACACTTAACAGGTGCTGCTTCTGAAATGCTAGTAGCCTACAAATTTACCAAAGCTGGGTACACTGTCTATTACCCTATGCTGACTc                                                                                                                                                                                                                                       >  1:128330/1‑301 (MQ=255)`  
                                                                            `ggACTTTGCGTACATGGCATGGGTACATGATGAAATCCAAGTAGGCTGCCGTACCGAAGAGATTGCTCAGGTGGTCATTGAGACCGCACAAGAAGCGATGCGCTGGGTTGGAGACCACTGGAACTTCCGGTGTCTTCTGGATACCGAAGGTAAGATGGGACCTAATTGGGCGATTTGCCACTGATACAGGAGGCTACTCATGAACGAAAGACACTTAACAGGTGCTGCTTCTGAAATGCTAGTAGCCTACAAATTTACCAAAGCTGGGTACACTGTCTA‑TACCCCATGCTGACTcctttct                                                                                                                                                                                                                                 >  1:116317/1‑295 (MQ=255)`  
                                                                             `gACTTTGCGTACATGGCATGGGTACATGATGAAATCCAAGTAGGCTGCCGTACCGAAGAGATTGCTCAGGTGGTCATTGAGACCGCACAAGAAGCGATGCGCTGGGTTGGAGACCACTGGAACTTCCGGTGTCTTCTGGATACCGAAGGTAAGATGGGTCCTAATTGGGCGATTTGCCACTGATACAGGAGGCTACTCATGAACGAAAGACACTTAACAGGTGCTGCTTCTGAAATGCTAGTAGCCTACAAATTTACCAAAGCTGGGTACACtctgtctcttatatacatctccgagcccc                                                                                                                                                                                                                                 >  1:346239/1‑273 (MQ=255)`  
                                                                               `cTTTGCGTACATGGCATGGGTACATGATGAAATCCAAGTAGGCTGCCGTACCGAAGAGATTGCTCAGGTGGTCATTGAGACCGCACAAGAAGCGATGCGCTGGGTTGGAGACCACGGGAACTTCCGGAGTCTTCTGGATACCGAAGGTAAGTTGGGTCCTAATTGGGCGATTTGCCACTGATACAGGAGGGTACTCATGAACGAAAGACACTTAACAGGTGCTGCTTCTGAAATGCTAGTAGCCTACAAATTTACCAAAGCTGGGTACACTGTCTATTACCCTATGCTGACTCAGAGTaaa                                                                                                                                                                                                                               >  1:70917/1‑301 (MQ=255)`  
                                                                                `caggCGTACATGGCATGGGTACATGATGAAATCCAAGTAGGCTGCCGTACCGAAGAGATTTCTCAGGTGGTCATTGAGACCGCACAAGAAGCGATGCGCTGGGTTGGAGACCACTGGAACTTCCGGTGTCTTCTGGATACCGAAGGTAAGATGGGTCCTAATTGGGCGATTTGCCACTGATACAGGAGGCTACTCATGAACGAAAGACACTTAACAGGTGCTGCTTCTGAAATGCTAGTAGCCTACAAATTTACCAAAGCTGGGTACACTGTCTATTACCCTATGCTGACTCAGAGTAAag                                                                                                                                                                                                                              <  1:225235/298‑1 (MQ=255)`  
                                                                                 `ttGCGTACATGGCATGGGTACAGGATGAAATCCAAGTAGGCTGCCGTACCGAAGAGATTGCTCAGGTGGTCATTGAGACCGCACAAGAAGCGATGCGCTGGGTTGGAGACCACTGGAACGTCCGGTGTCTTCTGGATACCGAAGGTAAGATGGGTCCTAATTGGGAGATTTGCCACTGATACAGGAGGCTACTCATGAACGAAAGACACTTAACAGGTGCTGCTTCTGAAATGCTAGTAGCCTACAAATTTACCAAAGCTGGGTACACTGTCTATTACCCTATGCTGACTCAGAGTAAaga                                                                                                                                                                                                                             <  1:342583/301‑1 (MQ=255)`  
                                                                                 `aagagacagtggcatggGTACATGATGAAATCCAAGTAGGCTGCCGTACCGAAGAGATTGCTCAGGTGGTCATTGAGACCGCACAAGAAGCGATGCGCTGGGTTGGAGACCACTGGAACTTCCGGTGTCTTCTGGATACCGAAGGTAAGATGGGTCCTAATTGGGCGATTTGCCACTGATACAGGAGGCTACTCATGAACGAAAGACACTTAACAGGTGCTGCTTCTGAAATGCTAGTAGCCTACAAATTTACCAAAGCTGGGTACACTGTCTATTACCCTATGCTGACTCAGAGTAAaga                                                                                                                                                                                                                             <  1:76898/292‑1 (MQ=255)`  
                                                                                  `tGCGTACATGGCATGGGTACATGATGAAATCCAAGTAGGCTGCCGTACCGAAGAGATTGCTCAGGTGGTCATTGAGACCGCACAAGAAGCGATGCGCTGGGTTGGAGACCACTGGAACTTCCGGTGTCTTCTGGATACCGAAGGTAAGATGGGTCCTAATTGGGCGATTTGCCACTGATACAGGAGGCTACTCATGAACGAAAGACACTTAACAGGTGCTGCTTCTGAAATGCTAGTAGCCTACAAATTTACCAAAGCTGGGTACACTGTCTATTAcccctgtctcttatacacatctccc                                                                                                                                                                                                                            >  1:137485/1‑279 (MQ=255)`  
                                                                                   `gCGTACATGGCATGGGTACATGATGAAATCCAAGTAGGCTGCCGTACCGAAGAGATTGCTCAGGTGGTCATTGAGACCGCACAAGAAGCGATGCGCTGGGTTGGAGACCACTGGAACTTCCGGTGTCTTCTGGATACCGAAGGTAAGATGGGTCCTAATTGGGCGATTTGCCACTGATACAGGAGGCTACTCATGAACGAAAGACACTTAACAGGTGCTGCTTCTGAAATGCTAGTAGCCTACAAATTTACCAAAGCTGGGTACACTGTCTATTACCCTATGCTGACTCAGAGTAAAGAgg                                                                                                                                                                                                                           <  1:231945/301‑1 (MQ=255)`  
                                                                                          `tggcatggGTACATGATGAAATCCAAGTAGGCTGCCGTACCGAAGAGATTGCTCAGGTGGTCATTGAGACCGCACAAGAAGCGATGCGCTGGGTTGGAGACCAATGGAACTTCCGGTGTCTTCTGGATACCGAAGGTAAGATGGGTCCTAATTGGGCGATTTGCCACTGATACAGGAGGATACTCATGAACGAAAGACACTTAACAGGTGCTGCTTCTGAAATGCTAGTAGCCTACAAATTTACCAAAGCTGGGTACACTGTCTATTACCCTATGCTGACTCAGAGTAAAGAGGACTTGGt                                                                                                                                                                                                                    <  1:57600/301‑1 (MQ=255)`  
                                                                                          `tcggagatgtgtataagagacagcAAGTAGGCTGCCGTACCGAAGAGATTGCTCAGGTGGTCATTGAGACCGCACAAGAAGCGATGCGCTGGGTTGGAGACCACTGGAACTTCCGGTGTCTTCTGGATACCGAAGGTAAGATGGGTCCTAATTGGGCGATTTGCCACTGATACAGGAGGCTACTCATGAACGAAAGACACTTAACAGGTGCTGCTTCTGAAATGCTAGTAGCCTACAAATTTACCAAAGCTGGGTACACTGTCTATTACCCTATGCTGACTCAGAGTAAAGAGGACTTGGt                                                                                                                                                                                                                    <  1:168715/278‑1 (MQ=255)`  
                                                                                            `gtataagagacagatgaaagccaagtaggCTGCCGTACCGAAGAGATTGCTCAGGTGGTCATTGAGACCGAACAAGAAGCGATGCGCTGGGTTGGAGACCACTGGAACTTCCGGTGTCTTCTGGATACCGAAGGTAAGATGGGTCCTAATTGGGCGATTTGCCACTGATACAGGAGGCTACTCATGAACGAAAGACACTTAACAGGTGCTGCTTCTGAAATGCTAGTAGCCTACAAATTTACCAAAGCTGGGTACACTGTCTATTACCCTATGCTGACTCAGAGTAAAGAGGACTTGGTTG                                                                                                                                                                                                                  <  1:125750‑M1/289‑1 (MQ=255)`  
                                                                                            `gcatggGTACATGATGAAATCCAAGTAGGCTGCCGTACCGAAGAGATTGCTCAGGTGGTCATTGAGACCGCACAAGAAGCGATGCGCTGGGTTGGAGACCACTGGAACTTCCGGTGTCTTCTGGATACCGAAGGTAAGATGGGTCCTAATTGGGCGATTTGCCACTGATACAGGAGGCTACTCATGAACGAAAGACACTTAACAGGTGCTGCTTCTGAAATGCTAGTAGCCTACAAATTTACCAAAGCTGGGTACACTGTCTATTACCCTATGCTGACTCAGAGTAAagactgtctcttat                                                                                                                                                                                                                  >  1:227961/1‑290 (MQ=255)`  
                                                                                            `gacagggtacaTGATGAAATCCAAGTAGGCTGGCGTACCGAAGAGATTGCTCAGGTGGTCATTGAGACCGCACAAGAAGCGATGCGCTGGGTTGGAGACCACTGGAACTTCCGGTGTCTTCTGGATACCGAAGGTAAGATGGGTCCTAATTGGGCGATTTGCCACTGATACAGGAGGCTACTCATGAACGAAAGACACTTAACAGGTGCTGCTTCTGAAATGCTAGTAGCCTACAAATTTACCAAAGCTGGTTACACTGTCTATTACCCTATGCTGACTCAGAGTAAAGAGGACTTGGTTG                                                                                                                                                                                                                  <  1:106822‑M1/297‑1 (MQ=255)`  
                                                                                                `gggTACATGATGAAATCCAAGTAGGCTGCCGTACCGAAGAGATTGCTCAGGTGGTCATTGAGACCGCACAAGAAGCGATGCGCTGGGTTGGAGACCACTGGAGCTTCCGGTGTCTTCTGGATACCGAAGGTAAGTTGGGTCCTAATTGGGCGATTTGCCACTGATACAGGAGGCTACTCATGAACGAAAGACACTTAACAGGTGCTGCTTCTGAAATGCTAGTAGCCTACAAATTTACCAAAGCTGGGTACACTGTCTATTACCCTATGCTGACTCAGAGTAAagctgtctcttatacaca                                                                                                                                                                                                              >  1:113402/1‑285 (MQ=255)`  
                                                                                                `gggTACATGATGAAATCCAAGTAGGCTGCCGTACCGAAGAGATTGCTCAGGTGGTCATTGAGACCGCACAAGAAGCGATGCGCTGGGTTGGAGACCACTGGAACTTCCGGTGTCTTCTGGATACCGAAGGTAAGATGGGTCCTAATTGGGCGATTTGCCACTGATACAGGAGGCTACTCATGAACGAAAGACACTTAACAGGTGCTGCTTCTGAAATGCTAGTAGCCTACAAATTTACCAAAGCTGGGTACACTGTCTATTACCCTATGCTGACTCAGAGTAAAGAGGACTTGGTTGTATG                                                                                                                                                                                                              >  1:269760‑M1/1‑301 (MQ=255)`  
                                                                                                 `ggTACATGATGAAATCCAAGTAGGCTGCCGTACCGAAGAGATTGCTCAGGTGGTCATTGAGACCGCACAAGAAGCGATGCGCTGGGTTGGAGACCACTGGAACTTCCGGTGTCTTCTGGATACCGAAGGTAAGATGGGTCCTAATTGGGCGATTTGCCACTGATACAGGAGGCTACTCATGAACGAAAGACACTTAACAGGTGCTGCTTCTGAAATGCTAGTAGCCTACAAATTTACCAAAGCTGGGTACACTGTCTATTACCCTATGCTGACTCAGAGTAAagactgtctcttatacaca                                                                                                                                                                                                             >  1:337417/1‑285 (MQ=255)`  
                                                                                                     `cATGATGAAATCCAAGTAGGCTGCCGTACCGAAGAGATTGCTCAGGTGGTCATTGAGACCGCACAAGAAGCGATGCGATGGGTTGGAGACCACTGGAACTTCCGGTGTCTTCTGGATACCGAACGTAAGATGGGTCCTAATTGGGCGATTTGCCACTGATACAGGAGGCTACTCATGAACGAAAGACACTTAACAGGTGCTGCTTCTGAAATGCTAGTAGCCTACAAATTTACCAAAGCTGGGTACACTGTCTATTACCCTATGCTGACTCAGAGTAAAGAGGACTTGGTTGTATGTAAgg                                                                                                                                                                                                         <  1:312765/301‑1 (MQ=255)`  
                                                                                                     `aatgatgAAATCCAAGTAGGCTGTCGTACCGAAGAGATTGCTCAGGTGGTCATTGGGACCGCACAAGAAGCGATGCGCTGGGTTGGAGACCACTGGAACTTCCGGTGTCTTCTGGATACCGAAGGTAAGATGGGTCCTAATTGGGCGATTTGCCACTGATACAGGAGGCTACTCATGAACGAAAGACACTTAACAGATGCTGCTTCTGAAATGCTAGTAGCCTACAAATTTACCAAAGCTGGGTACACTGTCTATTACCCTATGCTGACTCAGAGTAAAGAGGagggggggggagggaagg                                                                                                                                                                                                         <  1:174933/300‑18 (MQ=255)`  
                                                                                                        `gatgaAATCCAAGTAGGCTGCCGTACCGAAGAGATTTCTCAGGTGGTCATTGAGACCGCACAAGAAGCGATGCGCTGGGTTGGAGACCACTGGAACTTCCGGTGTCTTCTGGATACCGAAGGTAAGATGGGTCCTAATTGGGCGATTTGCCACTGATACAGGAGGCTACTCATGAACGAAAGACACTTAACAGGTGCTGCTTCTGAAATGCTAGTAGCCTACAAATTTACCAAAGCTGGGTACACTGTCTATTACCCTATGCTGACTCAGAGTAAAGAGGACTTGGTtgtatgtctgtctc                                                                                                                                                                                                      >  1:273164/1‑294 (MQ=255)`  
                                                                                                             `aaTCCAAGTAGGCTGCCGTACCGAAGAGATTGCTCAGGTGGTCATTGAGACCGCACAAGAAGCGATGCGCTGGGTTGGAGACCACTGGAACTTCCGGTGTCTTCTGGATACCGAAGGTAAGATGGGTCCTAATTGGGCGATTTGCCACTGATACAGGAGGCTACTCATGAACGAAAGACACTTAACAGGTGCTGCTTCTGAAATGCTAGTAGCCTACAAATTTACCAAAGCTGGGTACACTGTCTATTACCCTATGCTGACTCAGAGTAAAGAGGACTTGGTTGTATGTAAGGATGGTaaa                                                                                                                                                                                                 <  1:100589/301‑1 (MQ=255)`  
                                                                                                              `aTCCAAGTAGGCTGCCGTACCGAAGAGATTGCTCAGGTGGTCATTGAGACCGCACAAGAAGCGATGCGCTGGGTTGGAGACCACTGGAACTTCCGGTGTCTTCTGGATACCGAAGGTAAGATGGGTCCTAATTGGGCGATTTGCCACTGATACAGGAGGCTACTCATGAACGAAAGACACTTAACAGGTGCTGCTTCTGAAATGCTAGTAGCCTACAAATTTACCACAGCTGGGTACACTGTCTATTACCCTATGCTGACTCAGAGTAAAGAGGACTTGGTTGTATGgaaggattgatgct                                                                                                                                                                                                >  1:82759‑M1/1‑287 (MQ=255)`  
                                                                                                                     `tAGGCTGCCGTACCGAAGAGATTGCTCAGGTGGTCATTGAGACCGCACAAGAAGCGATGCGCTGGGTTGGAGACCACTGGAACTTCCGGTGTCTTCTGGATACCGAAGGTAAGATGGGTCCTAATTGGGCGATTTGCCACTGATACAGGAGGCTACTCATGAACGAAAGACACTTAACAGGTGCTGCTTCTGAAATGCTAGTAGCCTACAAATTTACCAAAGCTGGGTACACTGTCTATTACCCTATGCTGACTCAGAGTAAAGAGGACTTGGTTGTATGTAAGGATGGTAAATTTAGTaa                                                                                                                                                                                         <  1:218684/301‑1 (MQ=255)`  
                                                                                                                       `ggCTGCCGTACCGAAGAGATTGCTCAGGTGGTCATTGAGACCGCACAAGAAGCGATGCGCTGGGTTGGAGACCACTGGAACTTCCGGTGTCTTCTGGATACCGAAGGTAAGATGGGTCCTAATTGGGCGATTTGCCACTGATACAGGAGGCTACTCATGAACGAAAGACACTTAACAGGTGCTGCTTCTGAAATGCTAGTAGCCTACAAATTTACCAAAGCTGGGTACACTGTCTATTACCCTATGCTGACTCAGAGTAAAGAGGACTTGGTTGTATGTAAGGATGGTAAATTTAGTAAgg                                                                                                                                                                                       >  1:119396/1‑301 (MQ=255)`  
                                                                                                                               `ctgagatgtgtataagagacaggtcagtgagaccgcacaagaagcGATGCGCTGGGTTGGAGACCACTGGAACTTCCGGTGTCTTCTGGATACCGAAGGTAAGATGGGTCCTAATTGGGCGATTTGCCACTGATACAGGAGGCTACTCATGAACGAAAGACACTTAACAGGTGCTGCTTCTGAAATGCTAGTAGCCTACAAATTTACCAAAGCTGGGTACACTGTCTATTACCCTATGCTGACTCAGAGTAAAGAGGACTTGGTTGTATGgaaggattgatgacattcgtagtacgtacat                                                                                                                                                                               <  1:254531‑M1/280‑32 (MQ=255)`  
                                                                                                                                    `aaGAGATTGCTCAGGTGGTCATTGAGACCGCACAAGAAGCGATGCGCTGGGTTGGAGACCACTGGAACTTCCGGTGTCTTCTGGATACCGAAGGTAAGATGGGTCCTAATTGGGCGATTTGCCACTGATACAGGAGGCTACTCATGAACGAAAGACACTTAACAGGTGCTGCTTCTGAAATGCTAGTAGCCTACAAATTTACCAAAGCTGGGTACACTGTCTATTACCCTATGCTGACTCAGAGTAAAGAGGACTTGGTTGTATGgaaggattgatgacattcgtagtacgtacatcattt                                                                                                                                                                          <  1:265734‑M1/301‑37 (MQ=255)`  
                                                                                                                                       `agaTTGCTCAGGTGGTCATTGAGACCGCACAAGAAGCGATGCGCTGGGTTGGAGACCACTGGAACTTCCGGTGTGTTCTGGATACCGAAGGTAAGATGGGTCCTAATTGGGCGATTAGCTACTGATACAGGAGGCTACTCATGAACGAAAGACACTTAACAGGTGCTGTTTCTGAAATGCTAGAAGCCTACAAATTTACCAACGCTGGGTACACTGTCTATTACCCTATGCTGACTCAGAGTAAAGAGGACTTGGTTGTATGgcaggattgatgacatctgtagcacgtacataatttcgt                                                                                                                                                                       >  1:331246‑M1/1‑262 (MQ=255)`  
                                                                                                                                       `agaTTGCTCAGGTGGTCATTGAGACCGCACAAGAAGCGATGCGCTGGGTTGGAGACCACTGGAACTTCCGGTGTCTTCTGGATACCGAAGGTAAGATGGGTCCTAATTGGGCGATTTGCCACTGATACAGGAGGCTACTCATGAACGAAAGACACTTAACAGGTGCTGCTTCTGAAATGCTAGTAGCCTACACATTTACCAAAGCTGGGTACACTGTCTATTACCCTATGCTGACTCAGAGTAAAGAGGACTTGGTTGTATGgaaggattgatgacattcgtagtacgtacatcatttcgt                                                                                                                                                                       >  1:91378‑M1/1‑262 (MQ=255)`  
                                                                                                                                                 `ggtggtCATTGAGACCGCACAAGAAGCGATGCGCTGGGTTGGAGACCACTGGAACTTCCGGTGTCTTCTGGATACCGAAGGTAAGATGGGTCCTAATTGGGCGATTTGCCACTGATACAGGAGGCTACTCATGAACGACAGACACTTAACAGGTGCTGCTTCTGAAATGCTAGTGGCCTACAAATTTACCACAGCTGGGTACACTGTCTATTACCCTATGCTGACTCAGAGTAAAGAGGACTTGGTTGTATGTAAGGATGGTAAATTTAGTACGGTTCAGGTTAAAACAGCAACAACGGtt                                                                                                                                                             >  1:326656/1‑301 (MQ=255)`  
                                                                                                                                                  `gtggtCATTGAGACCGCACAAGAAGCGATGCGCTGGGTTGGAGACCACTGGAACTTCCGGTGTCTTCT‑GATACCGAAGGTAAGATGGGTCCTAATTGGGCGATTTGCCACTGATACAGGAGGCTACTCATGAACGAAAGACACTTAACAGGTGCTGCTTCTGAAATGCTAGTAGCCTACAAATTTACCAAAGCTGGGTACACTGTCTATTACCCTATGCTGACTCATAGTAAAGAGGACTTGGTTGTATGgacggattgatgacattcgtagtacgtacatcatttcgtgaggccattaag                                                                                                                                                           >  1:89094‑M1/1‑250 (MQ=255)`  
                                                                                                                                                    `ggtCATTGAGACCGCACAAGAAGCGATGCGCTGGGTTGGAGACCACTGGAACTTCCGGTGTCTTCTGGATACCGAAGGTAAGATGGGTCCTAATTGGGCGATTTGCCACTGATACAGGAGGCTACTCATGAACGAAAGACACTTAACAGGTGCTGCTTCTGAAATGCTAGTAGCCTACAAATTTACCAAAGCTGGGTACACTGTCTATTACCCTATGCTGACTCAGAGTAAAGAGGACTTGGTTGTATGgaaggattgatgacattcgtagtacgtacatcatttcgtgaggccattaagg                                                                                                                                                          >  1:197659‑M1/1‑249 (MQ=255)`  
                                                                                                                                                       `cATTGAGACCGCACAAGAAGCGATGCGCTGGGTTGGAGACCACTGGAACTTCCGGTGTCTTCTGGATACCGAAGGTAAGATGGGTCCTAATTTGGCGATTTGCCACTGATACAGGAGGCTACTCATGAACGAAAGACACTTAACAGGTGCTGCTTCTGAAATGCTAGTAGCCTACAAATTTACCAAAGCTGGGTACACTGTCTATTACCCTATGCTGACTCAGAGTAAAGAGGACTTGGTTGTATGgaaggattgatgacattcgtagtacgtacatcatttcgtgaggccattaaggaca                                                                                                                                                       >  1:221548‑M1/1‑246 (MQ=255)`  
                                                                                                                                                                  `tgggctcggagatgtggataagagacagccactggaacttccggtgtcttctggatacCGAAGGTAAGATGGGTCCTAATTGGGCGATATGCCACTGATACAGGAGGATACTCATGAACGAAAGACACTTAACAGGTACTGCTTCTGAAATGCTAGTAGCCTACAAATTTACCAAAGCTGGGTACACTGTCTATTACCCTATGCTGACTCAGAGTAAAGAGGACTTGGTTGTATGgaaggattgatgacattcgtagtacgtacatcatttcgtgaggccattaaggacatgcacgaagag                                                                                                                                            <  1:73870‑M1/273‑67 (MQ=255)`  
                                                                                                                                                                          `gCGATGCGCTGGGTTGGAGACCACTGGAACTTCCGGTGTCTTCTGGATACCGAAGGTAAGATGGGTCCTAATTGGGCGATTTGCCACTGATACAGGAGGCTACTCATGAACGAAAGACACTTAACAGGTGCTGCTTCTGAAATGCTAGTAGCCTACAAATTTACCAAAGCTGGGTACACTGTCTATTACCCTATGCTGACTCAGAGTAAAGAGGACTTGGTTGTATGgaaggattgatgacattcgtagtacgtacatcatttcgtgaggccattaaggacatgcacgaagagtatgcaga                                                                                                                                    <  1:162785‑M1/301‑75 (MQ=255)`  
                                                                                                                                                                            `gATGCGCTGGGTTGGAGACCACTGGAACTTCCGGTGTCTTCTGGATACCGAAGGTAAGATGGGTCCTAATTGGGCGATTTGCCACTGATACAGGAGGCTACTCATGAACGAAAGACACTTAACAGGTGCTGCTTCTGAAATGCTAGTAGCCTACAAATTTACCAAAGCTGTGTACACAGTCTATTACCCTATGCTGACTCAGAGTAAAGAGGACTTGGTGGTATGgaaggattgatgacattcgtagtacgtacatcatttcgtgaggccattaaggacatgcacgaagagtatgcagata                                                                                                                                  <  1:250663‑M1/301‑77 (MQ=255)`  
                                                                                                                                                                                   `ggggtTGGAGACCACTGGAACTTCCGGTGTCTTCTGGATACCGAAGGTAAGATGGGTCCTAATTGGGCGATTTGCCACTGATACAGGAGGCTACTCATGAACGAAAGACACTTAACAGGTGCTGCTTCTGAAATGCTAGTAGCCTACAAATTTACCAAAGCTGGGTACACTGTCTATTACCCTATGCTGACTCAGAGTAAAGAGGACTTGGTTGTATGgaaggattgatgacattcgtagtacgtacatcatttcgtgaggccattaaggacatgcacgaagagtatgcagataagaactc                                                                                                                           <  1:275714‑M1/300‑84 (MQ=255)`  
                                                                                                                                                                                    `gggTTGGAGACCACTGTAACTTCCGGTGTCTTCTGGATACCGAAGGTAAGATGAGTCCTAATTGGGCGATTTGCCACTGATACAGGAGGCTACTCATGAACGAAAGACACTTAACAGGTGCTGCTTCTGAAATGCTAGTAGCCTACAAATTTACCAAAGCTGGGTACACTGTCTATTACCCTATGCTGACTCAGAGTAAAGAGGACTTGGTTGTATGTAAGGATGGTAAATTTAGTAAGGTTCAGGTTAAAACAGCCACAACGGTTCAAACCAACACAGGAGATGCCAAGCAGGTTAGGCt                                                                                                                          <  1:277665/301‑1 (MQ=255)`  
                                                                                                                                                                                    `gggTTGGAGACCACTGGAACTTCCGGTGTCTTCTGGATACCGAAGGTAAGATGGGTCCTAATTGGGCGATTTGCCACTGATACAGGAGGCTACTCATGAACGAAAGACACTTAACAGGTGCTGCTTCTGAAATGCTAGTAGCCTACAAATTTACCAAAGCTGGGTACACTGTCTATTACCCTATGCTGACTCAGAGTAAAGAGGACTTGGTTGTATGgaaggattgatgacattcgtagtacgtacatcatttcgtgaggccattaaggacatgcacgaagctgtctcttatacacatctc                                                                                                                          >  1:39135‑M1/1‑217 (MQ=255)`  
                                                                                                                                                                                      `gTTGGAGACCACTGGAACTTCCGGTGTCTTCTGGATACCGAAGGTAAGATGGGTCCTAATTGGGCGATTTGCCACTGATACAGGAGGCTACTCATGAACGAAAGACACTTAACAGGTGCTGCTTCTGAAATGCTAGTAGCCTACAAATTTACCAAAGCTGGGTACACTGTCTATTACCCTATGCTGACTCAGAGTAAAGAGGACTTGGTTGTATGgaaggattgatgacattcgtagtacgtacatcatttcgtgaggccattaaggacatgcacggagagtatgcagataaggactcttt                                                                                                                        >  1:159687‑M1/1‑215 (MQ=255)`  
                                                                                                                                                                                           `agaCCACTGGAACTGCCGGTGTCTTCTGGATACCGAAGGTAAGATGGGACCTAATTGGGCGATTTGCCAATGATACAGGAGGCTACTCATGAACGAAAGACACTTAACAGGTGCTGCTTCTGAAATGCTAGTAGCCTACAAATATACCAAAGCTGGGTACACTGTCTATTACCCTATGCTGACTCAGAGTAAAGAGGACTTGGTTGTATGgaaggattgatgacattcgtagtacgtacatcatttcgtgaggccattaaggacatgcacgaagagtatgcagataaggactctttcaaac                                                                                                                   <  1:208668‑M1/301‑92 (MQ=255)`  
                                                                                                                                                                                               `cACTGGAACTTCCGGTGTCTTCTGGATACCGAAGGTAAGATGGGTCCTAATTGGGCGATTTGCCACTGATACAGGAGGCTACTCATGAACGAAAGACACTTAACAGGTGCTGCTTCTGAAATGCTAGTAGCCTACAAATTTACCAAAGCTGGGTACACTGTCTATTACCCTATGCTGACTCAGAGTAAAGAGGACTTGGTTGTATGgaaggattgatgacattcgtagtacgtacatcatttcgtgaggccattaaggacatgcacgaagagtatgcagataaggactctttcaaacaatc                                                                                                               >  1:80867‑M1/1‑206 (MQ=255)`  
                                                                                                                                                                                                   `ggAACTTCCGGTGTCTTCTGGATACCGAAGGTAAGATGGGTCCTAATTGGGCGATTTGCCACTGATACAGGAGGCTACTCATGAACGAAAGACACTTAACAGGTGCTGCTTCTGAAATGCTAGTAGCCTACAAATTTACCAAAGCTGGGTACACTGTCTATTACCCTATGCTGACTCAGAGTAAAGAGGACTTGGTTGTATGTAAGGATGGTAAATTTAGTAAGGTTCAGGTTAAAACAGCCACAACGGTTCAAACCAACACAGGAGATGCCAAGCAGGTTAGGCTAGGTGGATGCGGTAg                                                                                                           >  1:298072/1‑301 (MQ=255)`  
                                                                                                                                                                                                    `aatgtgtataagagacagggATACCGAAGGTAAGATGGGTCCTAATTGGGCGATTTGCCACTGATACAGGAGGCTACTCATGAACGAAAGACACTTAACAGGTGCTGCTTCTGAAATGCTAGTAGCCTACAAATTTACCAAAGCTGGGTACACTGTCTATTACCCTATGCTGACTCAGAGTCAAGAGGACTTGGTTGTATGTAAGGATGGTAAATTTAGTAAGGTTCAGGTTAAAACAGCCACAACGGTTCAAACCAACACAGGAGATGCCAAGCAGGTTAGGCTAGGTGGATGCGGTAgg                                                                                                          <  1:93123/283‑1 (MQ=255)`  
                                                                                                                                                                                                            `ggTGTCTTCTGGATACCGAAGGTAAGATGGGTCCTAATTGGGCGATTTGCCACTGATACAGGAGGCTACTCATGAACGAAAGACACTTAACAGGTGCTGCTTCTGAAATGCTAGTAGCCTACAAATTTACCAAAGCTAGGTACACTGTCTATTACCCTATGCTGACTCAGAGTAAAGAGGACTTGGTTGTATGgaaggattgatgacattcgtagtacgtacatcatttcgtgaggccattaaggacatgcacgaagagtatgcagataaggactctttcaaacaatctcctgcaacagta                                                                                                  >  1:141467‑M1/1‑193 (MQ=255)`  
                                                                                                                                                                                                               `gtCTTCTGGATACCGAAGGTAAGATGGGTCCTAATTGGGCGATTTGCCACTGATACAGGAGGCTACTCATGAACGAAAGACACTTAACAGGTGCTGCTTCTGAAATGCTAGTAGCCTACAAATTTACCAAAGCTGGGTACACTGTCTATTACCCTATGCTGACTCAGAGTAAAGAGGACTTGGTTGTATGgaaggattgatgacattcgtagtacgtacatcatttcgtgaggccattaaggacatgcacgaagagtatgcagataaggactctttcaaacaatctcctgcaacagtacgg                                                                                               >  1:286231‑M1/1‑190 (MQ=255)`  
                                                                                                                                                                                                               `gtCTTCTGGATACCGAAGGTAAGATGGGTCCTAATTGGGCGATTTGCCACTGATACAGGAGGCTACTCATGAACGAAAGACACTTAACAGGTGCTGCTTCTGAAATGCTAGTAGCCTACAAATTTACCAAAGCTGGGTACACTGTCTATTACCCTATGCTGACTCAGAGTAAAGAGGACTTGGTTGTATGgaaggattgatgacattcgtagtacgtacatcatttcgtgaggccattaaggacatgcacgaagagtatgcagataaggacctgtctcttatacacatctccgagcccacg                                                                                               >  1:132381‑M1/1‑190 (MQ=255)`  
                                                                                                                                                                                                                 `cttctGGATACCGAAGGTAAGATGGGTCCTAATTGGGCGATTTGACACTGATACAGGAGGCTACTCATGAACGAAAGACACTTAACAGGTGCTGCTTCTGAAATGCTAGTAGACTACAAATTTACCAAAGCTGGGTACACTGTCTATTACCCTATGCTGACTCAGAGTAAAGAGGACTTGGTTGTATGgaaggattgatgacattcgtagtacgtacatcatttcgtgaggccattaaggacatgcacgaagagtatgcagataaggactctttcaaacaatctcctgcaacagtacggga                                                                                             >  1:85153‑M1/1‑188 (MQ=255)`  
                                                                                                                                                                                                                  `tgtataagagacagggtaagatgggtcctaATTGGGCGATTTGCCACTGATACAGGAGGCTACTCATGAACGAAAGACACTTAACAGGTGCTGCTTCTGAAATGCTAGTAGCCTACAAATTTACCAAAGCTGGGTACACTGTCTATTACCCTATGCTGACTCAGAGTAAAGAGGACTTGGTTGTATGgaaggattgatgacattcgtagtatgtacatcatttcgtgaggccattaaggacatgcacgaagagtatgcagataaggactctttcaaacaatctcctgcaacagtacgggag                                                                                            <  1:239794‑M1/287‑115 (MQ=255)`  
                                                                                                                                                                                                                   `tctGGATACCGAAGGTAAGATGGGTCCTAATTGGGCGATTTGCCACTGATACAGGAGGCTACTCATGAACGAAAGACACTTAACAGGTGCTGCTTCTGAAATGCTAGTAGCCTACAAATTTACCAAAGCTGGGTACACTGTCTATTACCCTATGCTGACTCAGAGTAAAGAGGACTTGGTTGTATGTAAGGATGGTAAATTTAGTAAGGTTCAGGTTAAAACAGCCACAACGGTTCAAACCAACACAGGAGATGCCAAGCAGGTTAGGCTAGGTGGATGCGGTAGGTCCGAATATAAgctg                                                                                           >  1:199411/1‑298 (MQ=255)`  
                                                                                                                                                                                                                                `ggTAAGATGGGTCCTAATTGGGCGATTTGCCACTGATACAGGAGGCTACTCATGAACGAAAGACACTTAACAGGTGCTGCTTCTGAAATGCTAGTAGCCTACAAATTTACCAAAGCTGGGTACACTGTCTATTACCCTATGCTGACTCAGAGTAAAGAGGACTTGGTTGTATGgaaggattgatgacattcgtagtacgtacatcatttcgtgaggccattaaggacatgcacgaagagtatgcagataaggactctttcaaacaatctcctgcaacagtacgggaggtgttctgatgtct                                                                              >  1:49512‑M1/1‑173 (MQ=255)`  
                                                                                                                                                                                                                                `ggTAAGATGGGTCCTAATTGGGCGATTTGCCACTGATACAGGAGGCTACTCATGAACGAAAGACACTTAACAGGTGCTGCTTCTGAAATGCTAGTAGCCTACAAATTTACCAAAGCTGGGTACACTGTCTATTACCCTATGCTGACTCAGAGTAAAGAGGACTTGGTTGTATGTAAGGATGGTAAATTTAGTAAGGTTCAGGTTAAAACAGCCACAACGGTTCAAACCAACACAGGAGATGCCAAGCAGGTTAGGCTAGGTGGATGCGGTAGGTCCGAATATAAGGATGgagctgtcgctt                                                                              >  1:329144/1‑292 (MQ=255)`  
                                                                                                                                                                                                                                `gacaggATTGGTCCTAATTGGGCGATTTGCCACTGATACAGGAGGCTACTCATGAACGAAAGACACTTAACAGGTGCTGCTTCTGAAATGCTAGTAGCCTACAAATTTACCAAAGCTGGGTACACTGTCTATTACCCTATGCTGACTCAGAGTAAAGAGGACTTGGTTGTATGTAAGGATGGTAAATTTAGTAAGGTTAAGGTTAAAACAGCCACAACGGTTCAAACCAACACAGGAGATGCCAAGCAGGTTAGGCTAGGTGGATGCGGTAGGTCCGAATATAAGGATGGAGACTTTGACa                                                                              <  1:103409/296‑1 (MQ=255)`  
                                                                                                                                                                                                                                 `gTAAGATGGGTCCTAATTGGGCGATTTGCCACTGATACAGGAGGCTACTCATGAACGAAAGACACTTAACAGGTGCTGCTTCTGAAATGCTAGTAGCCTACAAATTTACCAAAGCTGGGTACACTGTCTATTACCCTATGCTGACTCAGAGTAAAGAGGACTTGGTTGTATGgaaggattgatgacattcgtagtacgtacatcatttcgtgaggccattaaggacatgcacgaagagtatgcagatcaggactctttcaaacaatctcctgcaacagtacgggaggctgtctcttataca                                                                             >  1:21515‑M1/1‑172 (MQ=255)`  
                                                                                                                                                                                                                                                        `aTTTGCCACTGATACAGGAGGCTACTCATGAACGAAAGACACTTAACAGGTGCTGCTTCTGAAATGCTAGTAGCCTACAAATTTACCAAAGCTGGGTACACTGTCTATTACCCTATGCTGACTCAGAGTAAAGAGGACTTGGTTGTATGgaaggattgatgacattcgtagtacgtacatcatttcgtgaggccattaaggacatgcacgaagagtatgcagataaggactctttcaaacaatctcctgcaacagtacgggaggtgttctgatgtctgactacctgaaagtgctgcaagca                                                      >  1:206063‑M1/1‑149 (MQ=255)`  
                                                                                                                                                                                                                                                          `cagCCAATGATACAGGAGGCTACTCATGAACGAAAGACACTTAACAGGTGCTGCTTCTGAAATGCTAGTAGCCTACAAATTTACCAAAGCTGGGTACACTGTCTATTACCCTATGCTGACTCAGAGTAAAGAGGACTTGGTTGTATGTAAGGATGGTAAATTTAGTAAGGTTCAGGTTAAAACAGCCACAACGGTTCAAACCAACACAGGAGATGCCAAGCAGGTTAGGCTAGGTGGATGCGGTAGGTCCGAATATAAGGATGGAGACTTTGACATTCTTGCGGTTGTGGTTGACGAAGAt                                                    <  1:14784/299‑1 (MQ=255)`  
                                                                                                                                                                                                                                                                 `tGATACAGGAGGCTACTCATGAACGAAAGACACTTAACAGGTGCTGCTTCTGAAATGCTAGTAGCCTACCAATTTACCAAAGCTGGGTACACTGTCTATTACCCTATGCTGACTCAGAGTAAAGAGGACTTGGTTGTATGgaaggattgctgacattcgaagtacgtacatcatttcgtgaggacattaaggacatgcacgaagaggatgcagataaggactctttcaaacaatctcctgcaacagtacgggaggtgttctgatgtctgactacctgaaagtgctgcaagcaatcaaaagt                                             <  1:320500‑M1/301‑162 (MQ=255)`  
                                                                                                                                                                                                                                                                  `gATACAGGAGGCTACTCATGAACGAAAGACACTTAACAGGTGCTGCTTCTGAAATGCTAGTAGCCTACAAATTTACCAAAGCTGGGTAAACTGTCTATTACCCTATGCTGACTCAGAGTAAAGAGGACTTGGTTGTATGgaaggattgatgacattcgtagtacgtacatcatttcgtgaggccattaaggacatgcacgaagagtatgcagataaggactctttcaaacaatctcctgcaacagtacgggaggtgttctgatgtctgactacctgaaagtgctgcaagcaatcaaaagtt                                            >  1:135152‑M1/1‑139 (MQ=255)`  
                                                                                                                                                                                                                                                                     `aCAGGAGGCTACTCATGAACGAAAGACACTTAACAGGTGCTGCTTCTGAAATGCTAGTAGCCTACAAATTTACCAAAGCTGGGTACACTGTCTATTACCCTATGCTGACTCAGAGTAAAGAGGACTTGGTTGTATGTAAGGATGGTAAATTTAGTAAGGTTCAGGTTAAAACAGCCACAACGGTTCAAACCAACACAGGAGATGCCAAGCAGGTTAGGCTAGGTGGATGCGGTAGGTCCGAATATAAGGATGGAGACTTTGACATTCTTGCGGTTGTGGTTGACGAAGATGTGCTTAttct                                         >  1:237299/1‑299 (MQ=255)`  
                                                                                                                                                                                                                                                                        `ggaggCTACTCATGAACGAAAGACACTTAACAGGTGCTGCTTCTGAAATGCTAGTAGCCTACAAATTTACCAAAGCTGGGTACACTGTCTATTACCCTATGCTGACTCAGAGTAAAGAGGACTTGGTTGTATGgaaggattgatgacattcgtagtacgtacatcatttcgtgaggccattaaggacatgcacgaagagtatgcagataaggactctttcaaacaatctcctgcaacagtacgggaggtgttctgatgtctgactacctgaaagtgctgcaagcaatcaaaagttgcccta                                      >  1:340244‑M1/1‑133 (MQ=255)`  
                                                                                                                                                                                                                                                                          `aggCTACTCATGAACGAAAGACACTTAACAGGTGCTGCTTCTGAAATGCTAGTAGCCTACAAATTTACCAAAGCTGGGTACACTGTCTATTACCCTATGCTGACTCAGAGCAAAGAGGACTTGGTTGTATGTAAGGATGGTAAATTTAGTAAGGTTCAGGTTAAAACAGCCACAACGGTTCAAACCAACACAGGAGATGCCAAGCAGGTTAGGCTAGGTGGATGCGGTAGGTCCGAATATAAGGATGGAGACTTTGACATTCTTGCGGTTGTGGTTGACGAAGATGTGCTTATTTTCACAt                                    <  1:234078/301‑1 (MQ=255)`  
                                                                                                                                                                                                                                                                           `ggCTACTCATGAACGAAAGACACTTAACAGGTGCTGCTTCTGAAATGCTAGTAGCCTACAAATTTACCAAAGCTGGGTACACTGTCTATTACCCTATGCTGACTCAGAGTAAAGAGGACTTGGTTGTATGgaaggattgatgacattcgtagtacgtacatcatttcgtgaggccattaaggacatgcacgaagagtatgcagataaggactctttcaaacaatctcctgcaacagtacgggaggtgttctgatgtctgactacctgaaagtgctgcaagcaatcaaaagttgccctaaga                                   >  1:151699‑M1/1‑130 (MQ=255)`  
                                                                                                                                                                                                                                                                             `cTACTCATGAACGAAAGACACTTAACAGGTGCTGCTTCTGAAATGCTAGTAGCCTACAAATTTACCAAAGCTGGGTACACTGTCTATTACCCTATGCTGACTCAGAGTAAAGAGGACTTGGTTGTATGgaaggattgatgacattcgtagtacgtacatcatttcgtgaggccattaaggacatgcacgaagagtatgcagataaggactctttcaaacaatctcctgcaacagtacgggaggtgttctgatgtctgactacctgaaagtgctgcaagcaatcaaaagttgccctaagact                                 >  1:245662‑M1/1‑128 (MQ=255)`  
                                                                                                                                                                                                                                                                                    `gtataagagacagcTTAACAGGTGCTGCTTCTGAAATGCTAGTAGCCTACAAATTTACCAAAGCTGGGTACACTGTCTATTACCCTATGCTGACTCAGAGTAAAGAGGACTTGGTTGTATGTAAGGATGGTAAATTTAGTAAGGTTCAGGTTAAAACAGCCACAACGGTTCAAACCAACACAGGAGATGCCAAGCAGGTTAGGCTAGGTGGATGCGGTAGGTCCGAATATAAGGATGGAGACTTTGACATTCTTGCGGTTGTGGTTGACGAAGATGTACTTATTTTCACATGGGACGAAGt                          <  1:110757/288‑1 (MQ=255)`  
                                                                                                                                                                                                                                                                                    `agAACGAAAGACACTTAACAGGTGCTGCTTCTGAAATGCTAGTAGCCTACAAATTTACCAAAGCTGGGTACACTGTCTATTACCCTATGCTGACTCAGAGTAAAGAGGACTTGGTTGTATGTAAGGATGGTAAATTTAGTAAGGTTCAGGTTAAAACAGCCACAACGGTTCAAACCAACACAGGAGATGCCAAGCAGGTTAGGCTAGGTGGATGCGGTAGGTCCGAATATAAGGATGGAGACTTTGACATTCTTGCGGTTGTGGTTGACGAAGATGTGCTTATTTTCACATGGGACGAAGt                          <  1:144682/300‑1 (MQ=255)`  
                                                                                                                                                                                                                                                                                           `aaGACACTTAGCAGGAGCTGCGTCTGAAATGCTAGTAGCCTACAAATTTACCAAAGCTGGGTACACTGTCTATTACCCTATGCGGACTAAGAGTAAAGAGGACTTGGTTGTATGTAAGGATGGTAAATTTAGGAAGGTTCAGGTTAAAACAGCCACAACGGTTCAAACCAACACAGGAGATGCAAAGCAGGTTAGGCTAGGTGGATGCGGTAGGTCCGAATATAAGGATGGAGACTTTGACATTCTTGCGGTTGTGGTTGACGAAGATGTGCTTATTTTCACATGGGACGAAGTAAAAGGt                   <  1:212785/301‑1 (MQ=255)`  
                                                                                                                                                                                                                                                                                           `aaGACACTTAACAGGTGCTGCTTCTGAAATGCTAGTAGCCTACAAATTTACCAAAGCTGGGTACACTGTCTATTACCCTATGCTGACTCAGAGTAAAGAGGACTTGGTTGTATGgaaggattgatgacattcgtagtacgtacatcatttcgtgaggccattaaggacatgcacgaagagtatgcagataaggactctttcaaacaatctcctgcaacagtacgggaggtgttctgatgtctgactacctgaaagtgctgcaagcaatcaaaagttgccctaagactttccagtccaacta                   <  1:154636‑M1/301‑188 (MQ=255)`  
                                                                                                                                                                                                                                                                                               `cacTTAACAGGTGCTGCTTCTGAAATGCTAGTAGCCTACAAATTTACCAAAGCTGGGTACACTGTCTATTACCCTATGCTGACTCAGAGTAAAGAGGACTTGGTTGTATGTAAGGATGGTAAATTTAGTAAGGTTCAGGTTAAAACAGCCACAACGGTTCAAACCAACACAGGAGATGCCAAGCAGGTTAGGCTAGGTGGATGCGGTAGGTCCGAATATAAGGATGGAGACTTTGACATTCTTGCGGTTGTGGTTGACGAAGATGTGCTTATTTTCACATGGGACGAAGTAAAAGTTAAGa               >  1:291182/1‑301 (MQ=255)`  
                                                                                                                                                                                                                                                                                                `acTTAACAGGTGCTGCTTCTGAAATGCTAGTAGCCTACAAATTTACCAAAGCTGGGTACACTGTCTATTACCCTATGCTGACTCAGAGTAAAGAGGACTTGGTTGTATGTAAGGATGGTAAATTTAGTAAGGTTCAGGTTAAAACAGCCACAACGGTTCAAACCAACACAGGAGATGCCAAGCAGGTTAGGCTAGGTGGATGCGGTAGGTCCGAATATAAGGATGGAGACTTTGACATTCTTGCGGTTGTGGTTGACGAAGATGTGCTTATTTTCACATGGGACGAAGTAAAAGGTAAGAc              <  1:288418/301‑1 (MQ=255)`  
                                                                                                                                                                                                                                                                                                 `cTTAACAGGTGCTGCTTCTGAAATGCTAGTAGCCTACAAATTTACCAAAGCTGGGTACACTGTCTATTACCCTATGCTGACTCAGAGTAAAGAGGACTTGGTTGTATGgaaggattgatgacattcgtagtacgtacatcatttcgtgaggccattaaggacatgcacgaagagtatgcagataaggactctttcaaacaatctcctgcaacagtacgggaggtgttctgatgtctgactacctgaaagtgctgcaagcaatcaaaagttgccctaagactttccagtccaactatgtacg             >  1:4243‑M1/1‑108 (MQ=255)`  
                                                                                                                                                                                                                                                                                                 `cTTAACAGGTGCTGCTTCTGAAATGCTAGTAGCCTACAAATTTACCAAAGCTGGGTACACTGTCTATTACCCTATGCTGACTCAGAGTAAAGAGGACTTGGTTGTATGgaaggattgatgacattcgtagtacgtacatcatttcgtgaggccattaaggacatgcacgaagagtatgcagataaggactctttcaaacaatctcctgcaacagtacgggaggtgttctgatgtctgactacctgaaagtgctgcaagcaatcaaaagttgccctaagactttccagtccaactatgtacg             >  1:165467‑M1/1‑108 (MQ=255)`  
                                                                                                                                                                                                                                                                                                 `cTTAACAGGTGCTGCTTCTGAAATGCTAGTAGCCTACAAATTTACCAAAGCTGGGTACACTGTCTATTACCCTATGCTGACTCAGAGTAAAGAGGACTTGGTTGTATGgaaggattgatgacattcgtagtacgtacatcatttcgtgaggccattaaggacatgcacgaagagtatgcagataaggactctttcaaacaatctcctgcaacagtacgggaggtgttctgatgtctgactacctgaaagtgctgcaagcaatcaaaagttgccctaagactttccagtccaacgatgtacg             >  1:44818‑M1/1‑108 (MQ=255)`  
                                                                                                                                                                                                                                                                                                 `cTTAACAGGTGCTGCTTCTGAAATGCTAGTAGCCTACAAATTTACCAAAGCTGGGTACACTGTCTATTACCCTATGCTGACTCAGAGTAAAGAGGACTTGGTTGTATGgaaggattgatgacattcgtagtacgtacatcatttcgtgaggccattaaggacatgcacgaagagtatgcagataaggactctttcaaacaatctcctgcaacagtacgggaggtgttctgatgtctgactacctgaaagtgctgcaagcaatcaaaagttgccctaagacctgtctcttatacacatctcc             >  1:300918‑M1/1‑108 (MQ=255)`  
                                                                                                                                                                                                                                                                                                 `cTTAACAGGTGCTGCTTCTGAAATGCTAGTAGCCTACAAATTTACCAAAGCTGGGTACACTGTCTATTACCCTATGCTGACTCAGAGTAAAGAGGACTTGGTTGTATGgaaggattgatgacattcgtagtacgtacatcatttcgtgaggccattaaggacatgcacgaagagtatgcagataaggactctttcaaacaatctcctgcaacagtacgggaggtgttctgatgtctgactacctgaaagtgctgcaagcaatcaaaagttgccctaagacctgtctcttatacacatctcc             >  1:283007‑M1/1‑108 (MQ=255)`  
                                                                                                                                                                                                                                                                                                   `tAACAGGTGCTGCTTCTGAAATGCTAGTAGCCTACAAATTTACCAAAGCTGGGTACACTGTCTATTACCCTATGCTGACTCAGAGTAAAGAGGACTTGGTTGTATGgaaggattgatgacattcgtagtacgtacatcatttcgtgaggccattaaggacatgcacgaagagtatgcagataaggactctttcaaacaatctcctgcaacagtacgggaggtgttctgatgtctgactacctgaaagtgctgcaagcaatcaaaagttgccctaagactttccagtccaactatgtacgga           <  1:334580‑M1/301‑196 (MQ=255)`  
                                                                                                                                                                                                                                                                                                     `aCAGGTGCTGCTTCTGAAATGCTAGTAGCCTACAAATTTACCAAAGCTGGGTACACTGTCTATTACCCTATGCTGACTCAGAGTAAAGAGGACTTGGTTGTATGgaaggattgatgacattcgtagtacgtacatcatttcgtgaggccattaaggacatgcacgaagagtatgcagacaaggactctttcaaacaatctcctgcaacagtacgggaggtgttctgatgtctgactacctgaaagtgctgcaagcaatcaaaagttgccctaagactttccagtccaactatgtacggaac         <  1:3712‑M1/301‑198 (MQ=255)`  

                                                                                                                                                                                                                                                                                                            `|`                                                                                                                                                                                                                                                                                                              
`CTCTCATCTGCAAACTGTGGATTATCAAGACCGAAGAGATGCTCGTAGAGAAAGGCTTGAAGCATGGCTGGGATGGGGACTTTGCGTACATGGCATGGGTACATGATGAAATCCAAGTAGGCTGCCGTACCGAAGAGATTGCTCAGGTGGTCATTGAGACCGCACAAGAAGCGATGCGCTGGGTTGGAGACCACTGGAACTTCCGGTGTCTTCTGGATACCGAAGGTAAGATGGGTCCTAATTGGGCGATTTGCCACTGATACAGGAGGCTACTCATGAACGAAAGACACTTAACAGGTGCTGCTTCTGAAATGCTAGTAGCCTACAAATTTACCAAAGCTGGGTACACTGTCTATTACCCTATGCTGACTCAGAGTAAAGAGGACTTGGTTGTATGTAAGGATGGTAAATTTAGTAAGGTTCAGGTTAAAACAGCCACAACGGTTCAAACCAACACAGGAGATGCCAAGCAGGTTAGGCTAGGTGGATGCGGTAGGTCCGAATATAAGGATGGAGACTTTGACATTCTTGCGGTTGTGGTTGACGAAGATGTGCTTATTTTCACATGGGACGAAGTAAAAGGTAAGACATCCATGTGTGT  >  NC_001604.1/16208‑16808`

  

Alignment Legend

Aligned base mismatch/match (shaded by quality score): `ATCG/ATCG` `< 3 ≤` `ATCG/ATCG` `< 15 ≤` `ATCG/ATCG` `< 27 ≤` `ATCG/ATCG` `< 36 ≤` `ATCG/ATCG` `< 38 ≤` `ATCG/ATCG`

Unaligned base: `atcg`    Masked matching base: `atcg`    Alignment gap: `‑`    Deleted base: `‑`

Reads not counted as support for junction

read\_name Not counted due to insufficient overlap past the breakpoint.

read\_name Not counted due to not crossing MOB target site duplication.
